# Pandemic and Social Work: Exploring Finnish Social Workers’ Experiences through a SWOT Analysis

**DOI:** 10.1093/bjsw/bcab052

**Published:** 2021-03-29

**Authors:** Timo Harrikari, Marjo Romakkaniemi, Laura Tiitinen, Sanna Ovaskainen

**Affiliations:** Department of Social Work, University of Lapland, Rovaniemi, Finland

**Keywords:** adaptive governance, COVID-19 pandemic, diaries, glocal social work, resilience, SWOT-analysis

## Abstract

This article addresses the experiences of Finnish frontline social workers during the first wave of the COVID-19 pandemic in the spring of 2020. Two questions are addressed. First, ‘what types of challenges social work professionals faced’ in their everyday, ‘glocal’ pandemic setting and, second, what types of solutions they developed to meet these challenges. The data consist of 33 personal diaries that social work professionals created from mid-March to the end of May 2020. The diaries are analysed by a thematic content analysis and placed within the framework of a strengths, weaknesses, opportunities and threats analysis. The results suggest that the pandemic challenged social work at all levels, from face-to-face interactions to its global relations. The pandemic revealed not only the number of existing problems of social work, but also created new types of challenges. It demanded ultimate resilience from social workers and a new type of adaptive governance from social welfare institutions.

## Introduction

In early 2020, the coronavirus spread across the globe. Its impact on the economy, politics and daily life has been unprecedented. The global scale of the pandemic is complex in nature because the interdependence of the planet has dramatically increased over the past 30 years (Gomez *et al.*, 2013; Ductor and Leiva-Leon, 2016). The coronavirus pandemic can be called a ‘black swan’, as defined by the complexity theory (Taleb, 2010). The term refers to a sudden and unexpected series of events with far-reaching and long-lasting consequences. The current pandemic is expected to fundamentally change ‘the socioecological fabric of human communities and societies’; that is, it could change how societies organise themselves, how social institutions work and how people act in everyday settings.

The current article addresses the experiences of Finnish frontline social workers during the first wave of the coronavirus pandemic in the spring of 2020. We explore ‘what types of challenges social work professionals faced’ in their everyday ‘glocal’ (Harrikari and Rauhala, 2019; Livholts and Bryant, 2017) pandemic setting and what types of solutions they developed to meet these challenges. The data consist of 33 personal diaries that social work professionals created from mid-March to the end of May 2020. The diaries are analysed using a thematic content analysis and placed within the framework of a strengths, weaknesses, opportunities and threats (SWOT) analysis. The goal is to parse out the strengths and weaknesses, showing the opportunities and threats that social work faced in the first wave of the pandemic setting.

## Social work, complexity and adaptive governance

The coronavirus pandemic is a new type of phenomenon. It is true that epidemics have occurred throughout world history, but the current complexity of societal phenomena and their mutual interdependence manifest something new. Global economic interdependence, air travel and digital, real-time communication have increased drastically over the past three decades. In recent months, we have obtained information about the changes in the pandemic from television, social media and mobile phone feeds. This all indicates an irreversible compression of the world, where the global interconnectedness of events becomes intense and their local effects become unpredictable. (Harrikari and Rauhala, 2019).

The black swan event has proven to be a very complex phenomenon to control because it is emergent in nature. Even if the viral outbreak could be controlled through epidemiological means, the multiplier effects of the pandemic on other sectors of society, such as the economy and politics, may be unpredictable. All of this requires a systemic-oriented view, in which the changes of the socioecological fabric of society are analysed and appraised holistically, outlining the importance of the emergence, interconnectedness and interdependence of events (Boyd and Folke, 2012). The governance of this fabric requires an understanding of those measures by which the social resilience and adaptive governance of social institutions and professionals working in them can be promoted.

In 2021, we have obvious perceptions of how the socioecological fabric of Western countries has begun to organise themselves in a new way because of the pandemic, referred to as a ‘new normal’ by several governments. Obviously, there will be definitions of a ‘new deviance’ leading to a new kind of stigmatisation because of the outbreak response. Governmental restrictions affect the whole population, but they have the harshest impact on those marginalised individuals who already have difficulties in coping with their daily lives. The first international empirical analyses regarding the pandemic period suggest that, for example, those with a personal history of mental health problems may find those mental disorders exacerbated during a pandemic (e.g. Lee *et al.*, 2020). In March 2021, the case of India shows how the demographically richest but economically the poorest areas are equally vulnerable to resist human emergencies (Therborn, 2013).

## Pandemic and social work

Even if there is plenty of experience in social work practice and research on working in human emergencies and natural disasters, the global pandemic is a novel kind of experience for social work professionals and organisations. In terms of empirical research, knowledge production related to the pandemic is only just beginning (see, however, e.g. Finn Diderichsen *et al.*, 2019). The status of social work as a mandatory emergency profession has varied in countries during the first wave of the pandemic (Dominelli *et al.*, 2020). The primary public concerns presented in the media have addressed the availability of health care resources and the functioning of business and industry. The voices of frontline social work professionals are rarely heard in the public debate over the pandemic.

Social problems tend to become more difficult in times of crisis, but crises can also generate qualitatively new challenges. This tendency applies to social work as well. In recent months, social work has raised concerns regarding the homeless during lockdown, the loneliness of people with mental health problems in isolation and hidden problems in child protection, to name a few. Even though social work will play a significant role in the actions after a pandemic, during the ‘first wave’ of the current pandemic, social work has faced significant challenges, being forced to adopt ‘a new type of adaptive governance’ (Boyd and Folke, 2012), that is, new arrangements and innovations in the ways to provide social care for those in need. The examples mentioned above demonstrate how social work is increasingly surrounded by multidirectional dynamics, where social systems are complex, emergent and stratified. The ethical principles of social work are constantly being tested, and pressures to deviate from them tend to intensify in exceptional situations. Thus, although the well-being of health care staff has been the primary concern in the current pandemic, it is worth seriously considering social workers’ workload, well-being and resilience (McFadden *et al.*, 2015; Rapeli *et al.*, 2018).

## Methodological setting of the study: context, data, ethics and method

Finland, a country with six million inhabitants, is one of the Nordic welfare states. Within social policy studies, the Nordic countries enjoy a common reputation for their social democratic welfare regimes (Esping-Andersen, 1990). Over the past 30 years, however, Finland has been exposed to the same international trends of neoliberal governance and neoconservative policies as elsewhere (e.g. Harvey, 2005). Despite these developments, the traditional Nordic welfare ethos has survived rather well (Kvist *et al.*, 2012).

Since World War II, Finland has avoided large-scale catastrophes and societal crises, except for the deep economic recession of the 1990s. However, this situation changed when the coronavirus epidemic started in mid-March 2020. At the time, the Finnish government presented its first concerns towards the viral outbreak. Viral infections spread rapidly, and as a result, a state of emergency was declared in mid-April 2020. Cafes and restaurants were forced to close their doors. Schools and colleges switched to remote teaching. Several public services responsible for maintaining the well-being and social security of citizens were closed for visiting and moved to remote interactions. A significant governmental measure in the domestic policy was closing the border of the Uusimaa province on March 28 as the number of viral infections increased rapidly. Naturally, Finland became a part of global stagnation as national borders closed, air traffic faded away and people stopped travelling.

The closing of society took place in April to May 2020 and lasted three weeks. Since mid-May 2020, the government has planned to open up Finnish society gradually. First, the elementary schools were opened in mid-May because there was significant societal concern about the hidden problems of families. The colleges and higher education institutions still organised their teaching remotely. Cafes and restaurants were opened with new instructions for keeping distance between people and the regular use of hand disinfectants. At the end of May, 6,826 virus infections were confirmed in Finland (Tiitinen *et al.*, 2020). Summer was expected to reduce viral infections, but autumn of 2020 was expected to bring the second wave of the pandemic. In October 2020, the number of virus infections in Finland reached about the same level as in March 2020.

The starting point of this study is mid-March 2020, when the first serious concerns towards the international epidemic were brought forward. We submitted a diary writing request for social work professionals on 19 March 2020 in a closed Facebook group consisting of more than 3,000 members. As a consequence, 56 social work professionals signed up to write the diary. Professionals were asked to write about three issues: (i) ‘What kinds of observations do you have about the challenges that occur in the lives of social work clients during the pandemic?’, (ii) ‘What challenges do social work and its practices face during the pandemic?’ and (iii) ‘What kind of thoughts does the pandemic period evoke in you as a social work professional?’ The authors were informed that the form of the diaries would be free, and they were only asked to mark the date on their entries. The diaries were requested to be recorded until the end of May 2020.

In terms of research ethics and ethical reviewing, the research team followed the guidelines of the Finnish National Board on Research Integrity (TENK, 2019, p. 47–67). The participants were informed that writing a diary would be voluntary and sending it to the research team means giving one’s consent. The background information gathered was limited; only their age, gender, professional title and education, current working title and the main client groups with whom the participant was currently working were asked. Because the participants wrote their diaries as individuals and licenced social work practitioners, not as representatives of their institutions, no institutional background information was collected. In addition, the participants were reminded of their duty of confidentiality under the Social Welfare Act. If the diaries contained any identifiable information, they were anonymised by the researchers. However, the participants were reminded that situations encountered in everyday work can be described on varying abstract levels.

At the end of May 2020, the participants were asked to complete the diary work and send the diary to the research group. Finally, 33 diaries were returned to the encrypted project e-mail. Here, 94,139 total words were collected. Although the diaries could be written in any format, all the texts were sent in a digital format. There would have been conditions to continue writing because the pandemic was still continuing, but the research team wanted to stick to the original writing request.

There are several methodological options for analysing the diaries. One option would have been, for example, reading diaries as narratives and presenting a process-based description of the first wave of a pandemic. However, we chose a SWOT analysis as an analytical framework to obtain a multilayered view of a complex phenomenon. A SWOT analysis has been regarded as a useful planning tool for a situational analysis for programme evaluation and quality improvement. It was originally developed for business planning, and the goal was to identify information regarding strategic options of the firm in the competitive environment. Nowadays, this form of analysis is regarded as an appropriate strategic tool for many types of settings (e.g. Helms and Nixon, 2010).

Basically, the goal of a SWOT analysis is to capture the ‘internal’ strengths and weaknesses of a collective agent, which are controllable and can be acted upon (e.g. staff turnover). On the other hand, opportunities and threats are uncontrollable factors that form the external environment within which the agent operates (Chermack and Kasshanna, 2007). In the current article, however, the SWOT analysis is not applied to any specific ‘organisation’ as such but social work as a social and professional institution in a broad sense. Moreover, the information received from social workers’ diaries is mediated by nature and can only indicate their perceptions of the changes of social work in a pandemic setting.

Thus, exploring four fields through various combinations and forming new dimensions may offer options for the adaptive governance of social work. The analysis of the social workers’ conceptions was extended to all levels of activity, from client work to working teams and to institutional practices and interinstitutional activities. As a consequence, we decided to apply a thematic content analysis, where we could extract diary entries on the SWOT of social work in a pandemic setting. In the following, we explore the social resilience and adaptive governance of social work through a SWOT analysis, starting with the strengths and weaknesses and then moving on to the opportunities and threats that the pandemic opened up for social work.

## Strengths

From our data, we extract five key findings regarding the strengths of social work during the first phase of the pandemic: (i professional reflexivity and flexibility, (ii) rapid adaptation to change, (iii) tenacious professional ethics, (iv) the use of professional knowledge and competencies and (v) the promotion of structural social work, even in times of crisis.

In a new situation, where no ready-made ‘rules of the game’ existed, it was necessary to clarify the ongoing situation and develop a new kind of operating culture. Throughout our data, we noticed social workers’ considerable ability for ‘reflection and flexibility’ in the ways they worked. The pandemic period has been learning ‘new rules of the game’ not only in social work, but in all other societal activities:

‘Commonly agreed practices among our team have been launched very quickly, that is, how to maintain work with clients and services offered as stable as possible. Basically, we decided to organise all client meetings by phone, and a common criterion for meeting clients face-to-face or visiting their homes were created. – – Our team eventually decided to deliver a short corona-related information call to all the clients’*.* (D22/20/03/18)

In many places, the described reflexivity and flexibility led to *a* ‘rapid adaptation to the new situation and professional learning procedures’. Some social workers were informed of their proactive efforts among multiprofessional teams. They emphasised the complex nature of the pandemic and recalled its impact on, among others, food aid, people’s income and family systems. There were several entries in the diaries on these efforts. For example, a social worker working in health care asked her superior to send a bulletin where all staff members were reminded of the wide variety of influences of viral outbreak (D25/20/03/18). Another author claimed that social workers’ holistic societal views would be a strength through which they could anticipate the long-term effects of the crisis. In particular, many authors focused on the forthcoming autumn of 2020:

‘It seems clear to me that discussions, both among employees and with clients, are currently focused on the next fall and how all things are going to work then. Workers have been wondering whether there will be a pandemic peak next autumn, as there are now fewer mandatory child protection notices’. (D4/20/05/12)

The data samples presented above show how social workers were forced into ‘moral reasoning’ in the midst of their daily work. This is not, however, anything new because social workers’ daily lives used to consist of repeated professional decisions where professional ethics are endogenously present. However, the changing status of the viral outbreak, the challenges that it brought with it and the organisational instructions delivered to employees challenged social workers to ‘consider and apply ethical principles in their decision making’. This was one of the cross-cutting themes in several diaries. A single note of a social worker in her diary from the early stage of a pandemic reinforces the notion of how social work was understood as playing a stabilising role in clients’ lives: ‘My first thought: working with clients must continue as normally as possible. As the pandemic progresses, anxiety among clients will surely grow, their life situations become more difficult’ (D6/20/03/19). With this in mind, teams started to organise their everyday practices to take into account the new situation. In terms of preventing viral infections, teams were divided into two sections, allowing them to be present every other week in the workplace. Moreover, teams and workers sought to ensure the functioning of remote connections, the flow of information and the management of acute situations in clients’ lives (D6/20/03/19). The social workers’ strong commitment to professional ethical principles is evident in the following sample, where there is concern about children’s adequate food intake while schools were closed:

‘I was worried about the food issue for families with children, and I was in contact with the principal, the municipal social welfare director, and the finance director. However, my concern was ignored—Then I contacted the local parish and—they started immediately to organise food aid for families with children.—I offered even myself a helping hand for potato peeling’. (D27/20/03/19)

In the writings of the social workers, the ethical principles of social work and workers’ moral reasoning were seamlessly linked with the question of the ‘professional competencies, working skills and knowledge base’ needed and applied in a new situation. It seems that in times of crisis, social workers must have specific competencies, such as exceptional flexibility, operational readiness for unusual events, problem-solving abilities, the skills to distinguish correct information from the incorrect information and rapid knowledge acquisition and its subsequent application to everyday practice. Moreover, attention was paid to work-related competencies, such as IT skills, in the context of shifting from face-to-face interactions to remote connections. These new issues raised the question of one of the basic competencies of a social worker, that is, interaction skills:

‘Today, I have been thinking of what types of professional requirements this very specific era settles for social workers. I feel that the requirements are primarily related to interaction skills and secondly to empathy and “reachability”, so that a client dares to ask for help in the event of a crisis.––As I see it, resilience and flexibility in changing situations in particular, as well as the skills to promote dialogue and ask open-ended questions, must be emphasised when clients are facing a whole new kind of challenge.—In addition, the skills of expressing confidence and being truly present at a distance are to be emphasised’. (D22/20/04/16)

Finally, in the midst of the crisis, serious efforts emerged to develop social work, disseminate information in organisations, improve the flow of information in public and influence policy makers. Some social workers were disappointed with the pandemic information shared by their employer, particularly on social media, which led them to take an active role in providing information. This was carried out by, among other things, making videos on the services intended for children and young people in social media, participating actively in social media debates, writing in newspapers and contacting MPs and the leading government officials concerning the challenges in a pandemic setting:

‘Today, I sent an e-mail to my familiar Ministry of Social and Health Affairs officials and informed them what the whole situation looks like in terms of digital work. I promised them that I could help the Ministry compile the guidelines, as the municipalities need them immediately’. (D29/20/03/30)

## Weaknesses

As might be expected, the pandemic period brought a wide range of challenges to social work and revealed its weaknesses. Most of the emerging problems had been known before, but the pandemic made them even more visible. New types of challenges emerged, too. The pandemic showed that (i) crisis management and communication in social welfare organisations is inadequate, (ii) the infrastructure enabling employees to work remotely is considerably limited. In addition, the pandemic period increased (iii) internal conflicts within working teams, exacerbated a fair division of labour and promoted the polarisation of working teams into ‘camps’. Concerns were also raised about (iv) the narrowing of the social workers’ professional discretion and the new pandemic-related tasks assigned to them.

The first wave of the pandemic made visible that preparing for ‘crisis management’ in social welfare institutions is rather inadequate. In terms of weaknesses, the diaries highlighted the inadequate resources of social work, heavy workload, labour shortages and personal turnover and lay-offs, especially during the early stages of the crisis. However, new types of problems become evident. At the beginning of the crisis, all instructions and protective equipment for social workers were missing. Physical operating environments and their potential for spreading infections fell under critical scrutiny. These included, for example, trend-setting open-air offices and hospitals:

‘I expected that when I come to the hospital, I would be instructed to meet patients only with protective equipment, as – it would be easy for me to spread the virus in my unawareness. However, this was not the case, social workers did not receive any guidance on using protective equipment. As I asked for my superior about it, he urged me to apply horse sense’. (D3/20/03/19)

As a part of crisis management, both internal and external ‘crisis communication’ in organisations regarding the changes ensued by the pandemic failed. Several authors described how information did not flow, drowned in a flood of information or that it was not found at all. The guidelines of internal communication concerning, for example, telecommuting, data security and organising client meetings, changed frequently; these guidelines were incomplete or inconsistent, leading to confusion and stress among the employees. In terms of external communication, the practices of informing clients about the changes in services varied. In particular, problems arose in multilingual communication as an increased need for interpreting and explaining changes for foreign language clients emerged. In some organisations, communicating with clients through social media was completely banned:

*‘*Rumours and other similar stuff are rambling, it is difficult to know what to believe as no information comes out from any official direction. No one can be trusted anymore’. (D18/20/03/31)

‘I have received dozens of emails every day; updates, decisions, instructions’. (D11/20/03/20)

‘The corona-related updates are not allowed.’(D13/20/05/19)

The pandemic setting posed new challenges for social work organisations because conventional face-to-face practices could not be applied because of the risk of viral infection. Consequently, organisations and their employees were obliged to shift to a remote telecommuting mode. Several participants stated how the ‘attitudes’ expressed by the managers towards telecommuting were ‘unfavourable’ and ‘infrastructure supporting remote working limited’. In places, telecommunicating was forbidden, appropriate equipment was lacking, and social workers had to fight for using this equipment. Because no organisational guidelines were available, the employees had to rely on their own individual judgement whether a face-to-face encounter was necessary:

‘The employee is left completely alone to consider which of the meetings is a necessary face-to-face encounter and which is not’. (D7/20/04/04)

‘We finally managed to fight through telecommuting opportunity terms, which are like from the 60s. For a maximum of two consecutive days, only the writing of a statement, not, for example, calls to client families, nor to the authorities—reports on remote working must be submitted with approximately the accuracy of toilet visits’. (D18/20/03/21)

When shifting from office conditions to implementing social work remotely, new kinds of challenges arose. In relation to clients, the most significant challenge for social workers was the skills of emotional work at a distance: how to interpret client situations and how to express empathy to clients. Other remote working-related challenges concerned the boundary conditions of interaction, such as the functionality of technical tools, Internet access and the use of client programmes. One of the social workers wrote how a client information system could not be used remotely at the same time with other computer applications. Working at home, she faced the challenges of confidentiality and working arrangements:

*‘*There are also a spouse and three children at home. I decide to drive to the seaside so that I can call clients. I've built for myself the office in our sauna—vpn connection is repeatedly lost, the client information system throws me out then and now—electric outlet locates outside sauna and the phone must be charged on above the washing machine, on the other side of the bathroom.—The image of the young man was so grainy that I didn’t even see him when he started crying during the meeting’. (D6/20/03/19)

In addition to administrative, managerial and technical weaknesses, the participants reported on the challenges of collaboration and communication and an increase in the internal tensions among work teams. We could name at least four watersheds according to which teams began to be divided into ‘camps’. There were (i) those who could shift to remote connections and those who could not (‘telecommuting’), (ii) those who actively tried to solve the problems that arose and those who waited for guidance from their superiors (‘proactivity’), (iii) those who sought to find new forms for client meetings and those who were sceptic to or opposed them (‘client encounters’) and (iv those who were able to protect themselves from the viral infections and those who could not (‘protection’).

Thus, the attitudes towards the epidemic, changes in working methods and forms of client meetings lead to tensions between the team members. At the very first stage of the viral outbreak, attention was paid more to the issues of occupational health and safety than to clients. Discussing client cases in remote team meetings was described as ‘superficial’, and responsible social workers might feel left alone addressing the problems of clients. Moreover, in joint team meetings, the employees were assigned an increasing amount of duties. The division of labour caused feelings of injustice, and in some places, this led to controlling work mates’ workload and questioning their work ethics. The transition to remote teleworking was criticised, and ‘a climate of protection’ was developed in the workplace:

‘I decide to attend a doctor’s meeting remotely. I face a critical feedback from other team members on “why in the world?”. The meeting will be attended by 15 people, so where does our view on the 10 [gathering] limit go? Two people repeat to me that the doctor is already remote, I say why I couldn’t be.—No collegial support, no common guidelines for work’. (D10/20/03/27)

‘Colleagues have very different views on [client] outdoor meetings. Some think they work very well, and others think they should not be organised at all’. (D4/20/04/08)

Furthermore, there emerged a great deal of external pressure towards social work. Several collaborative partners closed or reduced their services, leading to an increase in advocacy work. The administrative tasks for social work managers increased drastically for the sake of the uncoordinated administrative claims in reporting various corona effects, including, for example, the needs for and use of safety equipment, the number of staff present at the office or remotely and the overall change of staff workload.

Finally, two specific issues arising from the diaries can be highlighted. First, social workers were prescribed to take care of new basic tasks, such as cleaning and securing hygiene. Second, in places, social work was positioned as the last resort ‘institutional tailboard’, that is, providing psychosocial or teleworking support for other employee groups and their families during a pandemic:

*‘*Skype meeting for social workers around the hospital district. As a profession, we showed a green light to the occupational health and safety manager’s initiative to start a counselling and support line for hospital district employees for the duration of the corona. From there, staff can ask for help and support in “problems related to their own social situation” as well as problems of their own family, children and grandparents’. (D3/20/03/31)

## Opportunities

The first wave of the pandemic asked people to open their eyes to new issues and opportunities to reform social work practice. At least six perspectives can be listed: (i) viewing clients’ life situations in a new light, (ii) innovative ideas for reforming client work practices, (iii) employees’ strong endurance, (iv) new organisation of everyday social work through a hybrid model to promote the well-being of employees (v) new options for cooperation and (vi) organisational practices brought by the pandemic.

The main task of social work is to take care of the well-being of the most vulnerable and marginalised citizens in society. In their diaries, the social workers wrote multifaceted observations about changes in their clients’ life situations and ‘viewed them in a new light’. Amidst a wide range of concerns regarding the viral outbreak, there are observations that clients, even in a very difficult situation and without social worker’s support, had coped with the situation quite well. There are also entries that the client’s situation had even improved during the pandemic period:

‘It was strange to note that parents who had not been able to cope before without receiving family work, offering day care option or support person, took a break from all services and did very well’. (D4/20/03/26)

Second, the social workers had captured momentary glimpses in the middle of everyday life, opening up an ‘innovative idea’ of consolidating a new practice shaped by the corona era. Attention was paid to the simple facts, such as how telephone meetings often offered more time for writing notes or how online meetings with the mentally ill and home-isolating young people reached the client group better than face-to-face meetings. Because of the use of remote connections, the social workers had to consider and solve all kinds of issues:

‘I end up describing my own appearance to the client so that he can know who he’s talking to. Should all new clients be asked to draw their images on me? Or could we just deliver our photo to them’. (D6/20/03/30)

Beyond new opportunities, social workers’ ‘professional endurance and resilience’ cannot be ignored. A social worker’s job is to deal with any kind of event or phenomenon present. In addition to taking care of their clients, other employees in the organisation and their next of kin, social workers reported on their activities during a pandemic:

‘We have increased our preparedness because of the fact that the support/consultation phone that usually rings only infrequently will ring more, and we have to face things that we have not dealt with before’. (D5/20/03/27)

‘When I look now back at the past spring, I wonder how I have been able to make health care instructions for child welfare services. After all, I don’t have any understanding or education about health care. It makes me terrifying’. (D2/20/05/20)

‘This is learning along the way’. (D5/20/04/07)

It is described above how social workers talked about the wide range of professional challenges related to organising work and the functioning of working teams. At the same time, however, many of them described how their own ‘personal daily lives changed as their team shifted to a hybrid working model’, that is, working flexibly between the office or remotely. Some authors said that there was now time and energy, for example, to take exercise. Colleagues had mentioned slowing down the pace of work and opening options to take care of the arrears work. Fatigue-related sick leaves were also found to drop because of teleworking and especially at the end of the first wave of the pandemic; there are reflective entries in the diaries about how rather permanent arguments of the working team had become silly in light of the pandemic. Likewise, the opportunities for ‘collaboration between organisations’ emerged in a new light at the end of May 2020:

‘It took only a moment to shift to the telecommuting mode, but it is working surprisingly well. Most of us think that working is smoother and more efficient at home. –– If you happen to be a night owl, and prefer to work in the evening, how do you take care of your free time? Especially if the work is interesting and engaging.—I discussed with my colleagues that this exceptional situation would allow for a fruitful platform for closer cooperation with partners’. (D6/20/03/25)

Finally, regarding opening opportunities, some ‘new administrative and institutional practices’ were launched. Some municipalities started to follow corona-specific costs in their accounting. Even if technological applications overall received criticism, some new applications, such as ‘Videovisit’ and ‘MS Teams’, were launched. The secured use of some applications, such as WhatsApp, had been debated for a long time, but with the pandemic, they were finally permitted in client work. One of the social workers said that she organised the afternoon session for the family carers and was surprised by the huge number of participants. The carers had told her that participating was much easier remotely.

## Threats

In contrast to the opportunities that emerged, the diaries were featured by various threats raised during the pandemic. ‘The images of threat varied depending on the stage of the pandemic, and these threats emerged on many levels, from general and unspecified to concrete and detailed concerns’. Concerns were related regarding, among other things, clients’ escalating life situations, difficulties in accessing support and being excluded from services. Moreover, attention was paid to equality between clients, offering services to people exposed to the virus and clients hiding needs of assistance. The participants were worried about the effects of viral outbreaks in general and in the near future and also related to their work teams:

‘I hope I am not an asymptomatic carrier of the virus’. (D3/20/03/27)

‘It feels like this is only calm before the storm’. (D11/20/03/26)

‘No one goes to sit close to another—Faces are serious, the atmosphere is quiet. —There is fear in the air’. (D9/20/03/23)

However, ‘the most significant concerns were related to clients’ life situations’. A particular concern was directed at families with children and the elderly. The mental health problems of the elderly were believed to increase because of loneliness and isolation. The emergency centre (112) had received several calls from the elderly with suicidal thoughts, so the centre staff contacted a social worker. One of the authors noted that ‘isolating the elderly for the sake of protecting them will turn against them’ (D8/20/04/14). There were also new challenges for families with children and children placements because of the risk of viral infection. Some relatives refused to take care of the child either because of the general risks of infection or because they, usually grandparents, belonged to a high-risk group (D7/20/04/12). This perception reflects the wider ‘threat of stigmatising social work clients’ as a result of the pandemic:

‘The client tells me at meeting that people are fading around him on the bus and while he is walking down the street—this leads to a multiplying stigma’. (D6/20/05/06)

‘The parents refused to take a young person home because she was regarded `contaminated’. (D2/20/04/02)

In addition to the rising risks of client stigmatisation, the threats were access to services and the closure of services. It became difficult to refer clients to collaborative partners because there was only limited access to their services, or the services had been closed down. The social contacts provided by social services for clients were believed to advance all types of recovery. Isolation was presumed to lead to protracted rehabilitation processes, incapacity for work and, consequently, rising costs for society (D13/20/3/23). The delineation of services caused by the pandemic became evident in a concrete way:

‘We had to lock the doors of the family centre as drug users started to dwell in the lobby and toilets because of the corona crisis. We have never seen anything like this before’. (D2/20/03/31)

Finally, the social workers were obliged to exercise their discretion in the middle of a new kind of uncertainty. Several diaries raised concerns that professional discretion might fade away in the future, with social work turning bureaucratic work and social workers having to act against their professional ethics. In addition, the position of social work in the service system was a cause for concern:

‘It seems that social work is being overlooked in many places—I hope, however, that the importance of social work will be recognised and that it will be emphasised after such a situation’. (D1/20/03/18)

‘In the beginning, we were told that our work is the so-called receding work, that is, we can be transferred to other, more important tasks. This week, an e-mail circulated a survey asking about our other trainings and opportunities to move on to other positions. I did not answer’. (D9/20/04/06)

## Main empirical findings and matching four dimensions of SWOT-analysis

The analysis reveals how the appearance of sudden and powerful biological mechanisms challenged social work at all levels, from face-to-face interactions to its global relations. Indeed, the ‘black swan’ has been a ‘glocal’, complex and emergent phenomenon that demands resilience from social workers and a new type of adaptive governance from social welfare institutions, not only after the pandemic, but also all along the way during the pandemic. The pandemic revealed not only the number of existing problems of social work, but also initiated new types of challenges. The main empirical findings of the explorative analysis are presented in Table 1.

**Table 1.** SWOT analysis of social work during the first wave of the corona pandemic, spring 2020.

Environment/Activities	Positive	Negative
Internal	Strengths Professional reflexivity and flexibility Rapid adaptation to change Tenacious professional ethics and moral reasoning Use of professional knowledge and competencies The promotion of structural social work	Weaknesses Inadequate crisis management and communication Rigid attitudes of managers and limited infrastructure restraining employees from working remotely Increase in tensions within work teams Narrowing professional discretion The new pandemic-related tasks assigned by the administration
External	Opportunities Viewing clients’ life situations in a new light Innovative ideas for reforming client work practices Employees’ strong endurance New organisation of everyday social work through a hybrid model New options for cooperation and administrative practices	Threats General and concrete images of threat Clients’ escalating life situations and stigmatisation of clients; ‘new deviance’ The closure of services and delays in client processes Limitations of professional ethics and discretion in a new uncertainty The invisible status of social work in the service system Heavy workload and creeping new demands from outside

Hereby, we present a set of questions for improving the ‘pandemic social work’ and the resilience of social workers while developing the adaptive governance of social welfare based on our analysis. Exploring each of the four dimensions separately can offer us at least the following questions to be taken into account with future pandemics: (i) ‘how to maintain social workers’ individual resilience’, strong client-oriented professional ethics, skills and developmental-friendly mindset (S), (ii) ‘how to make opening opportunities’ with clients, collaborative agents and administration visible (O), (iii) ‘how to improve crisis infrastructure and management’ in social welfare organisations (W) and (iv) ‘how to avoid the future workload’ conditioned by external tasks as well as ‘the lack of collegial support’ (T) in a future pandemic setting.

Moreover, exploring the combinations of these four dimensions can offer a more systemic view of the questions of resilience and adaptive governance of social work. The most common perspectives to be noticed in a crisis tend to be how to decrease weaknesses of social work to avoid threats (W-T) or how arising threats materialise previously known weaknesses (T-W). However, we may also ask to what extent social work should use its strengths to reduce threats (S-T) and, further, how to utilise the strengths available to capitalise on the opportunities resulting from the pandemic (S-O). Still, questions remain regarding to what extent social work can harness the opening opportunities to consolidate its strengths (O-S) or how it can value the opportunities to eliminate weaknesses (O-W). These options can remain open for social work in future pandemics.

## Conclusion

In the current article, we have addressed the challenges of social work during the first wave of the coronavirus pandemic by exploring the diary entries of 33 Finnish frontline social workers. This study shows how social work institutions were completely unprepared for a situation like the pandemic but adapted to this new situation quickly and launched adequate ways of working (Boyd and Folke, 2012). The first wave of the pandemic was a burden for frontline social workers and put their well-being both at work and home to the test.

There is plenty of research on social work in human emergencies and disasters, but the global pandemic is a novel kind of phenomenon for social work. Thus, empirical knowledge production related to the pandemic is only just beginning. Our explorative analysis revealed several existing problems (e.g. workload and staff turnover) as well as new types of challenges (e.g. physical protection from the virus and remote interaction with clients) of the ‘pandemic’ social work, which can be addressed in future research. Our empirical analysis certainly has its limitations. The study has been implemented in the Finnish context (Rapeli *et al.*, 2018) and with qualitative methods, which should be kept in mind when interpreting the findings. There have been fewer viral infections in Finland than elsewhere, and the social service system is of a good international standard. However, social workers seem to have faced similar challenges around the globe (Dominelli *et al.*, 2020). Qualitative diary data, in turn, can provide an authentic but limited view of everyday social work practice. It should be kept in mind that the experiences of social workers and the episodes they describe in diaries are mediated in nature and that they focus only on the first wave of the pandemic.

In general, much has been learned about operating in pandemic conditions during the fall of 2020. Whereas the emergence of the pandemic was still a ‘weak signal’ at the end of 2019, after 2020, it is now a global megatrend. Thus, it is reasonable to believe that global crisis phenomena such as pandemics will frame future social work. It follows that the ecological perspective of social work (Matthies and Närhi, 2019) must be taken even more seriously in the future. COVID-19 has been a ‘glocal’ phenomenon, where social workers have been obliged to face the leading principle of glocalisation: ‘think globally, act locally’ (Livholts and Bryant, 2017; Harrikari and Rauhala, 2019). The pandemic has had different effects depending on the local socio-ecological fabric, and thus, the means of social resilience may vary (Boyd and Folke, 2012). However, after all, social work communities must be seen increasingly as part of a global village, one where the interdependencies of different parts of the world have become irreversible.
